# Telehealth Usability, Engagement Patterns, and Technical Infrastructure in Managing Noncommunicable Diseases Among Health Care Professionals in Brazil, Ghana, Honduras, and the United Kingdom: Multinational Cross-Sectional Study

**DOI:** 10.2196/64070

**Published:** 2026-06-12

**Authors:** Ken Brackstone, Roberta Lins Gonçalves, Adriana Silvina Pagano, Zilma Silveira Nogueira Reis, Seth Kwaku Afagbedzi, Lysien Ivania Zambrano, Tainá Costa Pereira Lopes, Sarah Almeida Cordeiro, Julia Macedo Nunes, Wagner Meira Jr, James Batchelor, Antonio Luiz Pinho Ribeiro

**Affiliations:** 1 University of Southampton, University Road, Southampton, England, SO171BJ, United Kingdom, 44(0)23 8059 5000; 2 Universidade Federal do Amazonas, Manaus, Amazonas, Brazil; 3 Universidade Federal de Juiz de Fora, Juiz de Fora, Minas Gerais, Brazil; 4 Universidade Federal de Minas Gerais, Belo Horizonte, Minas Gerais, Brazil; 5 University of Ghana, Accra, Greater Accra, Ghana; 6 National Autonomous University of Honduras, Tegucigalpa, Francisco Morazán Department, Honduras

**Keywords:** telehealth systems, usability, noncommunicable diseases, low- to middle-income countries, LMICs, high- to middle-income countries, HMICs, high-income countries, HICs

## Abstract

**Background:**

Noncommunicable diseases (NCDs) account for over 70% of global deaths, with hypertension and diabetes serving as major contributors. The COVID-19 pandemic disrupted traditional health care services for NCDs and highlighted telehealth as a crucial alternative. Telehealth—encompassing synchronous and asynchronous electronic communication to deliver clinical services remotely—can overcome geographical barriers and enhance patient engagement. However, telehealth usability among health care professionals (HCPs) remains under-studied across low-, middle-, and high-income countries.

**Objective:**

This study aimed to examine which telehealth engagement patterns, technical infrastructure factors, and user profiles were most strongly associated with usability among HCPs and to descriptively compare these across 4 diverse countries: Brazil (high- to middle-income country), Ghana (low- to middle-income country), Honduras (low- to middle-income country), and the United Kingdom (high-income country).

**Methods:**

A multinational cross-sectional survey was conducted with 290 HCPs across 4 countries. Participants completed the System Usability Scale and provided data on telehealth engagement (eg, frequency, duration, and number of systems used), technical infrastructure (connection stability and support satisfaction), and their user profile (demographics, job role, and training received). Descriptive statistics summarized these patterns and usability scores. Multiple linear regression with bootstrap-based sensitivity analyses identified factors associated with telehealth usability. Given the nonprobability design, no formal inferential comparisons were made between countries. Instead, observed patterns were reported descriptively.

**Results:**

Higher telehealth usability scores were associated with greater connection stability (*b*=5.06, 95% CI 3.06-7.05), higher satisfaction with online support information (*b*=5.02, 95% CI 3.27-6.75), more frequent use (*b*=3.05, 95% CI 1.36-4.73), longer duration of use (*b*=1.59, 95% CI 0.49-2.68), and being a physician by profession (*b*=3.82, 95% CI 0.23-7.40). Average usability scores were highest among users in Ghana (mean 79.75, SD 14.19) and the United Kingdom (mean 79.00, SD 14.71), followed by Brazil (mean 72.01, SD 14.62) and Honduras (mean 63.09, SD 15.57). According to System Usability Scale guidelines, scores corresponded to “good” usability for users in Ghana, the United Kingdom, and Brazil and were below the “good” threshold for users in Honduras. While most users in Ghana (97/111, 87.4%), Honduras (31/38, 81.6%), and Brazil (57/80, 70.4%) reported using only 1 telehealth system, two-thirds of UK users (40/60, 66.7%) reported using 2 or more systems. User profiles also varied; prepandemic use was highest in Ghana (84/111, 75.7%) and lowest in Honduras (7/38, 18.4%). Other engagement patterns across countries were reported.

**Conclusions:**

Telehealth usability is driven by technical infrastructure reliability, a robust online support infrastructure, and an “experience effect” from frequent and long-term engagement. Descriptive differences in engagement patterns and infrastructure highlight the need for tailored strategies to address setting-specific challenges. These are essential to optimize telehealth integration and improve health care outcomes for patients with NCDs worldwide.

## Introduction

Noncommunicable diseases (NCDs) are responsible for over 70% of deaths worldwide, with hypertension and diabetes being major contributors to NCD-related mortality, including cardiovascular disease and stroke [1]. NCDs are often chronic and progressive and require complex and continuous management of physiological, environmental, and behavioral factors [1-3]. Treatment costs for both patients and health services are high and place substantial economic pressure on health care systems [1-3]. Low- to middle-income countries (LMICs) experience higher NCD mortality rates than high-income countries (HICs) [1,4]. This is driven in part by a rapid epidemiological transition characterized by increased consumption of processed foods, reduced physical activity, and rapid urbanization alongside persistent exposure to tobacco and alcohol products [1]. In response, the United Nations 2030 Agenda for Sustainable Development committed to developing new approaches to reduce premature mortality from NCDs by one-third [5]. As a result, innovative and cost-effective solutions to improve care for NCDs are becoming increasingly prevalent. These include telehealth, mobile health apps, remote monitoring systems, and electronic health platforms that facilitate communication between patients and health care professionals (HCPs) [6,7].

While telehealth has existed for decades, the COVID-19 pandemic necessitated a rapid shift in its clinical application. This occurred after a large number of patients with NCDs were reportedly lost to follow-up due to the increased risk of illness and death associated with face-to-face consultations [8,9]. As a result, telehealth quickly transitioned from a supplementary service to an essential platform for HCPs to maintain contact with patients [10]. “Telehealth” is a broad term for health care that is delivered remotely by a variety of clinicians and HCPs. Patients and clinicians do not need to be located in the same clinic or hospital. Instead, HCPs can deliver a range of clinical services—health assessment, consultation, diagnosis, treatment, supervision, and information—to patients remotely regardless of geographic barriers [9,11,12]. Thus, telehealth can help patients become active and more efficient participants in their own health outcomes, particularly in underserved areas, and is used in the comfort, convenience, and safety of the patients’ own homes [13]. Studies have shown similar or better results with telehealth than with traditional care for some chronic conditions [14].

The range of telehealth modalities for HCPs includes synchronous use (eg, face-to-face interactions in real time, including video consultations and web-based videoconferencing) and asynchronous use (eg, email, SMS text messages, teledermatology via image transmission, remote monitoring, and electronic transmission of prescriptions to pharmacists), which can take place in clinical and nonclinical settings [4,11]. These varying engagement patterns represent different technical requirements and clinical workflows. However, to reap the optimal benefits of telehealth, it must be considered usable by both patients and HCPs [15-19]. Studies have shown that usability is an essential attribute of technology systems and is related to acceptability by end users [20-23]. Telehealth is sometimes viewed as disruptive and complex by HCPs, requiring specialized strategies to manage its use—particularly among specific user profiles, such as HCPs with limited telehealth experience or varying levels of prior training [6,16,17,19]. Furthermore, technical infrastructure can impair the usability and effectiveness of telehealth, such as system failures or connectivity issues [17]. Preliminary reports during the early stages of the pandemic indicated that HCPs’ reluctance to use telehealth technologies was one of the main barriers to implementation in clinical practice [17].

Prior to the onset of the COVID-19 pandemic, telehealth was mostly used in clinical settings across HICs and high- to middle-income countries (HMICs) [24]. Conversely, numerous LMICs have encountered and continue to encounter significant infrastructure-related challenges in adopting the technology [25]. Many individuals living in LMICs have restricted admittance to medical services, travel significant distances to receive medical care, and postpone medical care until they experience a health emergency [26,27]. However, since the pandemic, a number of primary care practices across HMICs and LMICs have been forced to implement telehealth in diverse geographical areas [6]. The rapid expansion of telehealth across LMICs has strengthened the potential of telehealth in delivering effective health care, particularly among patients with NCDs.

User acceptance remains an essential ingredient for maintaining telehealth use. Existing research has primarily evaluated telehealth usability from the patient perspective [15-19]. However, it is equally important to assess telehealth usability from the perspectives of HCPs—a topic much less studied [11,16,28]. A systematic review recently concluded that more studies should assess telehealth usability in LMICs to paint a broader picture of challenges faced using telehealth worldwide [28]. Thus, a comparison of the characteristics of telehealth use and usability perceptions across HMICs and LMICs can offer enormous contributions to global health by facilitating telehealth consolidation and assisting in expanding telehealth to countries where health service access is limited.

The overall aim of this study was to examine factors associated with telehealth usability among HCPs managing NCDs across diverse health care settings. Adopting an exploratory approach, we first sought to identify which telehealth engagement factors, technical infrastructure elements, and user profiles were most strongly associated with perceived system usability. We anticipated that technical reliability—specifically connection stability and satisfaction with support infrastructure—would be primary correlates of usability perceptions. Second, this study sought to describe how these engagement patterns and technical contexts varied across 4 countries representing distinct health care environments: Brazil (HMIC), Ghana (LMIC), Honduras (LMIC), and the United Kingdom (HIC). By identifying these factors, we aimed to provide evidence-based insights to optimize the technical and clinical integration of telehealth for NCD management worldwide.

## Methods

### Study Design

An international cross-sectional online survey was conducted in Brazil, Ghana, Honduras, and the United Kingdom. The survey was reported in accordance with the STROBE (Strengthening the Reporting of Observational Studies in Epidemiology) guidelines for cross-sectional studies (Checklist 1 [29]).

### Participants and Recruitment

A nonprobability convenience sampling approach was used to recruit HCPs from a range of health care settings, including hospitals, health centers, and clinics specializing in NCD management. We restricted the sample to HCPs working in primary care settings as the long-term management and monitoring of common NCDs such as hypertension and diabetes mostly takes place in primary care [30]. Furthermore, telehealth was widely implemented in these settings during the COVID-19 pandemic to support routine follow-up consultations [31]. Recruitment followed a systematic identification process via professional networks and institutional collaborations. Potential participants were contacted via email, telephone, or personal invitation by the project leaders in each country. Individuals were provided with a standardized information sheet regarding the study’s purpose before being sent the digital survey link via email. In Brazil, participants were contacted primarily in the state of Minas Gerais, a large inland state in southeastern Brazil. In Honduras, participants were contacted within Tegucigalpa, the capital city. In Ghana, participants were contacted in Accra, the capital city, located on the southern coast of the country. Finally, in the United Kingdom, participants were contacted in 6 counties from the northeast to the south of England. Data collection was conducted over a 9-month period between August 2022 and May 2023.

The target sample size was determined to provide a baseline for descriptive precision. We initially calculated a minimum requirement of 264 participants (n=66 per country) to estimate mean usability scores with 95% confidence and a 5% margin of error based on previously reported System Usability Scale (SUS) values (mean 71.4, SD 11.6) [31] and an estimated population of 500 HCPs. While this study used a nonprobability design not intended for formal inferential comparisons between countries, this recruitment target was established to ensure sufficient total volume for descriptive reporting by setting and to provide adequate statistical power for the multiple linear regression.

### Measures

#### Engagement Patterns

Participants first reported the number of telehealth systems they used in their current clinical practice (1, 2, or 3 or more) and identified the name of the system used most frequently. All subsequent questions referred to this primary system. Participants then stated their frequency of use (1=“every 2-3 months”; 4=“every day”) and duration of use (1=“0-3 months”; 7=“5+ years”). After receiving definitions of synchronous and asynchronous telehealth use, participants then rated how often they used each method (1=“never”; 5=“very often”). Finally, participants selected the telehealth features they used most regularly from a list of 10 (eg, exchange of or sending information to patients) and indicated the number of NCDs they treated via telehealth from a list of 20 (eg, hypertension and diabetes).

#### User Profiles

Participants indicated their professional background and digital experience. This included whether they received telehealth training (yes or no), whether they had used telehealth systems prior to the COVID-19 pandemic (yes or no), and whether they were required to be on clinical premises to use their telehealth system (yes or no). Finally, participants rated their perceived level of competence with digital tools (1=“very poor”; 5=“excellent”).

#### Telehealth Usability

Participants completed the SUS, a 10-item scale providing usability scores from 0 to 100 [32-34]. Participants indicated their agreement with 10 items (eg, “This telehealth system is easy to use”; 0=“strongly disagree”; 4=“strongly agree”). Scores were calculated by summing the individual item scores and multiplying by 2.5. Following established guidelines, scores of 60 to 69 were considered “moderate,” scores of 70 to 79 were considered “good,” and scores above 80 were considered “excellent.”

#### Technical Infrastructure

Participants rated 2 key infrastructural factors regarding their primary system: their satisfaction with available online help and support information (1=“strongly disagree”; 5=“strongly agree”) and the stability of their internet connection during a typical telehealth session (1=“very poor”; 5=“excellent”).

#### Demographic Variables

Participants indicated their gender, age (categorized as 24-39, 40-49, 50-59, or ≥60 years), and occupation (physician or nonphysician). In Ghana and Honduras, participants also reported their practice setting (rural or urban).

### Statistical Analysis

We first used descriptive statistics to summarize participant demographics. Next, we conducted multiple linear regression to identify factors associated with usability within the overall sample. Given the exploratory nature of this study, variables with a *P* value below .20 in univariate models were entered into the multiple linear regression model following previous recommendations [35]. Prior to analysis, we evaluated the assumptions of linear regression. Some predictors (eg, connection stability) were ordinal in nature and exhibited negative skew. As such, they were treated as continuous following established precedents for Likert-type scales and to maintain model parsimony [36]. To account for potential biases related to this assumption, we used bootstrap-based sensitivity analyses with 5000 resamples to ensure the robustness of our estimates against violations of normality. We evaluated multicollinearity using tolerance and the variance inflation factor; all tolerance values were within acceptable ranges (tolerance>0.6; variance inflation factor<2)

Given the nonprobability sampling design, we did not perform formal inferential comparisons between countries. Instead, we summarized telehealth usability, user profiles, telehealth engagement patterns, and technical infrastructure by country to observe descriptive patterns across different health care settings. Analyses were conducted using SPSS Statistics (version 30; IBM Corp).

### Ethical Considerations

Ethics approval was obtained by the respective institutional review boards in all participating countries: Brazil (Research Ethics Committee of the Federal University of Minas Gerais, Certificate of Submission of Ethical Review 56604122.5.0000.5149; 5.380.538), Honduras (Biomedical Research Ethics Committee of the National Autonomous University of Honduras 075-2022), Ghana (Ghana Health Service Ethics Review Committee 04/19/22), and the United Kingdom (Ethics and Research Governance Online ID: 72962 – University of Southampton). The survey protocol was previously published [37]. Study information was provided on the first page of the digital questionnaire, and all participants provided informed consent electronically by selecting a mandatory agreement box prior to commencing the survey. Participant data were fully anonymized at the point of collection to ensure privacy and confidentiality, with no personally identifiable information stored. Participation was entirely voluntary, and no financial or material compensation was provided.

## Results

### Demographics and User Profiles

Table 1 shows demographics and user profiles by country. A sample of 290 HCPs completed the survey (mean age 41.00, SD 7.45; range 24-65 years). Telehealth users from Ghana (n=111), Brazil (n=81), the United Kingdom (n=60), and Honduras (n*=*38) completed the survey. Most participants were men (150/290, 51.7%), aged 40 to 49 years (141/290, 48.6%), physicians by profession (201/290, 69.3%), and based at public hospitals (190/290, 65.5%) apart from users in Ghana, who were based mostly at private hospitals (75/111, 67.6%). In terms of practice setting, most participants in Ghana and Honduras reported working in urban areas (104/111, 93.7%, and 37/38, 97.4%, respectively).

**Table 1.** Demographics, user profiles, and telehealth engagement by country (categorical variables; N=290).

Characteristic	Total, n (%)	Brazil (n=81), n (%)	Ghana (n=111), n (%)	Honduras (n=38), n (%)	UK (n=60), n (%)
Gender					
Men	150 (51.7)	19 (23.5)	86 (77.5)	19 (50)	26 (43.3)
Women	140 (48.3)	62 (76.5)	25 (22.5)	19 (50)	34 (56.7)
Age (y)					
24-39	115 (39.7)	31 (38.3)	37 (33.3)	29 (76.3)	18 (30)
40-49	141 (48.6)	42 (51.9)	69 (62.2)	3 (7.9)	27 (45)
50-59	28 (9.7)	7 (8.6)	5 (5.5)	4 (10.5)	12 (20)
≥60	6 (2.1)	1 (1.2)	0 (0)	2 (5.3)	3 (5)
Job role					
Physician	201 (69.3)	14 (17.3)	105 (94.6)	38 (100)	44 (73.3)
Other	89 (30.7)	67 (82.7)	6 (5.4)	0 (0)	16 (26.7)
Sector^a^					
Public	190 (65.5)	73 (92.4)	36 (32.4)	30 (78.9)	51 (85)
Private	98 (33.8)	6 (7.6)	75 (67.6)	8 (21.1)	9 (15)
Used telehealth before the pandemic					
No	128 (44.1)	41 (50.6)	27 (24.3)	31 (81.6)	29 (48.3)
Yes	162 (55.9)	40 (49.4)	84 (75.7)	7 (18.4)	31 (51.7)
Training received					
No	88 (30.3)	19 (23.5)	12 (10.8)	21 (55.3)	36 (60)
Yes	202 (69.7)	62 (76.5)	99 (89.2)	17 (44.7)	24 (40)
Required to be on clinical premises^a^					
No	178 (61.4)	65 (81.3)	37 (33.3)	32 (84.2)	44 (74.6)
Yes	110 (37.9)	15 (18.7)	74 (66.7)	6 (15.8)	15 (25.4)
Telehealth systems used^a^					
1	205 (70.7)	57 (71.3)	97 (87.4)	31 (81.6)	20 (33.3)
2 or more	84 (29.0)	23 (28.7)	14 (12.6)	7 (18.4)	40 (66.7)

a Two of the 290 participants did not fully complete all measures (n=1 from Brazil and n=1 from the United Kingdom). Percentages reflect and include these missing participants; therefore, percentages for the variables indicated do not add up to 100%.

A higher proportion of participants in Ghana (84/111, 75.7%) reported using telehealth systems before the COVID-19 pandemic compared with participants in the United Kingdom (31/60, 51.7%), Brazil (40/81, 49.4%), and Honduras (7/38, 18.4%). Furthermore, a higher proportion of participants in Ghana (99/111, 89.2%) and Brazil (62/81, 76.5%) reported having received training for their selected telehealth systems compared to participants in Honduras (17/38, 44.7%) and the United Kingdom (24/60, 40%).

### Telehealth Usability, Engagement Patterns, and Technical Infrastructure

Table 2 shows means and SDs of telehealth usability, engagement patterns, and technical infrastructure by country. Descriptively, participants in Ghana (mean 79.75, SD 14.19; range 30-100) and the United Kingdom (mean 79.00, SD 14.71; range 32.50-100) reported higher average usability scores than those in Brazil (mean 72.01, SD 14.62; range 25-100) and Honduras (mean 63.09, SD 15.57; range 7.5-100; Table 2). According to SUS guidelines, scores from users in Ghana, the United Kingdom, and Brazil fell within the “good” range, whereas scores from users in Honduras fell below this threshold.

**Table 2.** Telehealth usability, engagement patterns, and technical infrastructure by country (continuous or ordinal variables; N=290).

	Total, mean (SD)	Brazil (n=81), mean (SD)	Ghana (n=111), mean (SD)	Honduras (n=38), mean (SD)	UK^a^ (n=60), mean (SD)
System usability (0-100)	75.25 (15.62)	72.01 (14.62)	79.75 (14.19)	63.09 (15.57)	79.00 (14.71)
Frequency of telehealth use (1-4)	3.15 (1.00)	3.00 (0.82)	3.50 (0.81)	1.97 (1.05)	3.43 (0.89)
Duration of telehealth use (1-7)	4.37 (1.62)	4.88 (1.84)	4.21 (1.36)	2.97 (1.44)	4.87 (1.26)
Frequency of synchronous use (1-5)	2.07 (1.25)	2.53 (1.32)	1.38 (0.89)	2.47 (1.22)	2.50 (1.16)
Frequency of asynchronous use (1-5)	3.62 (1.15)	3.03 (1.15)	4.00 (0.89)	2.79 (1.02)	4.23 (1.00)
Connection stability (1-5)	3.82 (0.79)	3.88 (0.80)	3.78 (0.71)	3.37 (0.94)	4.12 (0.69)
Satisfaction with online support information (1-5)	3.84 (0.81)	3.84 (0.89)	4.23 (0.65)	3.00 (1.12)	3.63 (0.99)
Number of telehealth systems used	3.09 (1.67)	2.68 (1.88)	3.33 (1.12)	2.63 (1.65)	3.48 (2.08)
Number of NCDs^b^ treated	4.28 (2.97)	2.88 (2.41)	4.57 (2.04)	4.11 (3.03)	5.75 (4.10)
Self-rated digital skills (1-5)	3.88 (0.81)	3.79 (0.83)	4.22 (0.59)	3.79 (0.84)	3.43 (0.85)

a UK: United Kingdom.

b NCD: noncommunicable disease.

Regarding engagement patterns, most participants in Ghana (97/111, 87.4%), Honduras (31/38, 81.6%), and Brazil (57/81, 70.4%) reported using only 1 telehealth system in their clinical roles, whereas 66.7% (40/60) of the participants in the United Kingdom reported using 2 or more. Descriptively, the frequency of telehealth use was most pronounced among participants in Ghana and the United Kingdom. While Ghanaian and UK participants reported a higher frequency of asynchronous telehealth use, Ghanaian participants reported the lowest nominal frequency of synchronous telehealth use. On average, UK participants reported managing the largest number of NCDs via telehealth (approximately 6 conditions) compared with participants in Brazil, Ghana, and Honduras.

In terms of technical infrastructure, participants in the United Kingdom reported the highest levels of connection stability, whereas Ghanaian participants reported the greatest satisfaction with available help and online support information.

### Factors Associated With Telehealth Usability

Table 3 presents the results of the multiple linear regression. Diagnostic residual plots and formal testing indicated a slight negative skew, primarily driven by high ratings for connection stability and support satisfaction. To address these departures from normality and ensure the reliability of our inferences, we performed bootstrap-based sensitivity analyses with 5000 resamples. These analyses yielded results nearly identical to those of the original ordinary least squares regression, which confirmed model robustness. The final regression model was statistically significant (*F*_12,276_=17.45; *P*<.001) and accounted for 40.7% of the variance in telehealth usability scores (adjusted *R*^2^*=*0.407). Higher telehealth usability scores were associated with greater connection stability (*b*=5.06, 95% CI 3.06-7.05; SE 1.01; β=0.26; *P*<.001), higher satisfaction with online support information (*b*=5.02, 95% CI 3.27-6.75; SE 0.88; β=0.30; *P*<.001), participants who were physicians by profession (*b*=3.82, 95% CI 0.23-7.40; SE 1.82; β=0.11; *P*=.04), more frequent telehealth use (*b*=3.05, 95% CI 1.36-4.73; SE 0.85; β=0.19; *P*<.001), and longer duration of telehealth use (*b*=1.59, 95% CI 0.49-2.68; SE 0.55; β=0.17; *P*=.004).

**Table 3.** Multiple linear regression model identifying factors associated with telehealth usability (N=290)^a^.

	Univariate model			Multiple linear regression model		
	*b*	*R* ^2^	*P* value	*b* (SE; 95% CI)	β	*P* value
Demographics						
Women (reference=men)	4.97	0.03	.007	2.17 (1.65; −1.08 to 5.42)	0.07	.19
Age	0.25	0.02	.04	−0.02 (0.10; −0.22 to 0.18)	−0.01	.87
Physician (reference=other)	2.71	0.01	.17	3.82 (1.82; 0.23 to 7.40)	0.11	.04
User features						
Training received (reference=no)	8.15	0.06	<.001	2.56 (1.69; −0.77 to 5.89)	0.08	.13
Telehealth use before the pandemic (reference=no)	8.24	0.07	<.001	−1.63 (1.80; −5.17 to 1.91)	−0.05	.37
Skills and competencies with digital tools	3.86	0.04	<.001	0.73 (0.99; −1.22 to 2.67)	0.04	.46
Telehealth use						
Frequency of use	6.34	0.16	<.001	3.05 (0.85; 1.36 to 4.73)	0.19	<.001
Duration of use	3.18	0.11	<.001	1.59 (0.55; 0.49 to 2.68)	0.17	.004
Number of NCDs^b^ treated	0.93	0.03	.002	0.22 (0.28; −0.32 to 0.77)	0.04	.42
Number of telehealth features used	0.81	0.01	.14	−0.25 (0.46; −1.15 to 0.66)	−0.03	.59
Stability of connection	8.77	0.20	<.001	5.06 (1.01; 3.06 to 7.05)	0.26	<.001
Satisfaction with online support information	8.18	0.24	<.001	5.02 (0.88; 3.27 to 6.75)	0.30	<.001

a The 3 columns for *b*, *R*2, and *P* value show predictors that entered the maximum model before variable selection (backward method). All variables had a *P* value of less than .20 in univariate analysis. The 2 columns for β and *P* value show a multiple linear regression containing all β and *P* values (*F*_12,276_=17.45; *P*<.001; adjusted R2=0.407).

b NCD: noncommunicable disease.

## Discussion

### Principal Findings

This exploratory study examined factors associated with telehealth usability among HCPs managing NCDs across 4 distinct health care settings. We aimed to identify how engagement patterns, technical infrastructure elements, and user profiles influenced perceived usability in these global contexts. Connection stability emerged as a primary correlate, suggesting that technical reliability is a foundational element of telehealth adoption. Unstable connections can disrupt communication and clinical workflows, which may undermine the rapport building necessary for NCD management [17]. These findings align with those of prior research identifying technological reliability as a critical determinant of usability [17,38]. Satisfaction with online support information also emerged as a strong correlate. While system training can be valuable, clear digital guidance may help HCPs better navigate telehealth platforms and manage ongoing support requirements. This provides supporting evidence on the value of a robust technological infrastructure in integrating telehealth systems into the care of patients with NCDs [28].

Furthermore, the association between usability and both the frequency and duration of telehealth use suggests an “experience effect,” consistent with established technology acceptance models [39]. Thus, usability may be a longitudinal skill developed through routine integration and increasing experience as users incorporate telehealth into clinical workflows. This experience effect could also explain why physicians reported higher usability as their clinical roles may require more frequent and varied engagement with platform functionalities for clinical decision-making. Overall, differences in clinical responsibilities and “on-the-job” experience may be central to how various user profiles evaluate telehealth systems.

Telehealth usability, engagement patterns, and technical infrastructure also varied descriptively by country. HCPs in Ghana, the United Kingdom, and Brazil reported “good” usability according to SUS guidelines, whereas those in Honduras reported “moderate” scores [32,34]. Descriptive findings suggest that this may be linked to lower frequency and duration of telehealth use, poorer connection stability, and lower satisfaction with telehealth support in certain settings. These variations may reflect differing levels of digital maturity and resource allocation across HIC, HMIC, and LMIC contexts. Such disparities highlight the relevance of considering local infrastructure when evaluating how HCPs interact with and perceive digital health tools.

### Country-Specific Engagement Patterns and System Integration

Beyond the correlates of usability, this study revealed distinct country-specific engagement patterns that illustrate how telehealth is integrated across different resource settings. Notable differences in telehealth engagement across the 4 countries provide context for understanding adoption in diverse health care settings. Most users in Ghana (97/111, 87.4%), Honduras (31/38, 81.6%), and Brazil (57/81, 71.3%) reported using only 1 telehealth system in their clinical roles compared to UK users (20/60, 33.3%), with 66.7% (40/60) of the latter reporting using 2 or more systems. These engagement patterns may reflect differences in telehealth infrastructure and availability of diverse telehealth solutions among HICs, HMICs, and LMICs. For example, they may reflect a more mature digital landscape in the United Kingdom, where clinicians use a “toolkit” of task-specific telehealth systems. Conversely, HCPs in lower-resourced settings may rely on single, more versatile systems for patient care. However, the high prevalence of prepandemic telehealth use (84/111, 75.7%) and training (99/111, 89.2%)—both key elements of the user profile in Ghana—suggests greater familiarity and integration. This indicates that LMICs such as Ghana leveraged telehealth to address health care delivery challenges before the pandemic.

Telehealth engagement was more frequent among Ghanaian and UK users, possibly reflecting more integrated or better-resourced systems. For example, most Ghanaian participants were based in urban, private hospitals, which generally possess superior technological infrastructure compared to public facilities [40]. Furthermore, the higher frequency of asynchronous engagement among Ghanaian and UK users suggests that these systems serve functions beyond real-time consultations [41]. Interestingly, Ghanaian users descriptively reported the lowest frequency of synchronous telehealth use, potentially reflecting infrastructural challenges such as unreliable internet connection that hinder real-time interactions [8]. While Ghanaian participants nominally reported lower connection stability than those in the United Kingdom, Honduras reported the lowest observed connection stability overall despite maintaining synchronous use rates similar to those reported by Brazil and UK users. This suggests that synchronous functionality may not be as deeply embedded in the technical infrastructure of certain LMICs. These findings should be interpreted alongside contextual factors as differences in infrastructure or engagement patterns may influence how HCPs evaluate connection stability. However, as participants were predominantly from urban settings with generally good connectivity, the results cannot determine whether perceived reliability reflects infrastructure constraints, specific engagement patterns, or other contextual factors.

Finally, the results demonstrated differences in telehealth use for NCD management across countries. Notably, UK users managed the highest number of NCDs (mean 5.75, SD 4.10), which was relatively higher than users in Brazil, Ghana, and Honduras. This discrepancy may reflect the United Kingdom’s advanced telehealth infrastructure and broader policy support, enabling more effective clinical integration [39]. Conversely, challenges in Brazil and Honduras—limited technological infrastructure and fewer financial resources—may limit the range of NCDs managed through telehealth platforms [42].

### Drivers of Telehealth Usability and Clinical Integration

These findings have several implications for the practice and policy of telehealth systems, extending the literature by examining usability across multiple income-level contexts and highlighting descriptive differences in infrastructure that influence user experience in HICs, HMICs, and LMICs. First, these findings align with literature emphasizing reliable internet connectivity for the success of telehealth initiatives [40]. In LMICs such as Ghana and Honduras, efforts to improve internet infrastructure are essential. Investments in robust connectivity may mitigate implementation challenges and enhance usability perceptions [8]. Furthermore, the findings underscore the need for targeted policies to bolster capabilities in lower-resourced settings. For example, improving telehealth systems to include better synchronous functionality may be highly relevant, particularly in African countries where synchronous functionality may not be as embedded. Addressing these disparities could help promote more equitable access to health care services and optimize the management of NCDs worldwide.

Next, these findings highlight the value of comprehensive online user support, including technical assistance and user manuals. Health care services should focus on developing and maintaining strong online support systems to assist HCPs in navigating telehealth platforms effectively, which could include technical instructions and advice on clinical workflow integration. Furthermore, as the frequency and duration of telehealth use were significantly associated with usability, encouraging consistent use through policy and practice may improve user experience. Health care services could consider integrating telehealth into routine clinical practice and providing incentives for regular use to foster familiarity and efficiency.

Tailored strategies addressing the specific challenges of each country appear foundational to successful implementation. For example, in LMICs with lower internet stability, asynchronous telehealth may be more viable than synchronous methods, as observed in Ghana. Therefore, targeted efforts to enhance telehealth usability are pivotal, including improving technological infrastructure and ensuring reliable support services. Addressing these barriers could support the adoption and effectiveness of telehealth systems across diverse health care landscapes, potentially contributing to improved health outcomes for patients with NCDs [40,43-45]. International collaborations and investments from HICs may play a valuable supporting role in these efforts.

### Limitations and Future Research

To our knowledge, this was the first study to explore factors associated with telehealth usability across HIC, HMIC, and LMIC contexts. However, several limitations exist. First, the sample sizes for each country were small and obtained using nonprobability sampling, which limited generalizability. Data were collected primarily from physicians in urban settings. Consequently, we could not account for broader systemic factors such as regional health care penetration (HCP-to-population ratios) or specific connection technologies (eg, fiber vs wireless). Future research should explore telehealth usability in larger, more diverse samples—specifically targeting rural populations and private sector settings where infrastructure constraints may be more pronounced.

Second, this study relied on cross-sectional self-reported data, which may be subject to response bias. Longitudinal and interventional studies following new users over time would provide deeper insights into adoption trends and predictors of usability, which are likely to vary according to country and other telehealth characteristics. Additionally, qualitative methods such as interviews would provide richer insights into HCPs’ perceived challenges. This would inform tailored strategies considering contextual differences—such as health care infrastructure and cultural attitudes toward telehealth—to facilitate successful integration for HCPs managing NCDs.

Finally, we used a binary response to establish baseline presence of telehealth training. This does not capture the depth, quality, or specific nature of such training, which can range from basic technical instruction to complex clinical workflow integration. The use of granular measures to distinguish between basic technical instruction and more comprehensive clinical workflow training is required. We also investigated usability through the SUS, which does not independently measure facets such as effectiveness, efficiency, security, or satisfaction [46], nor did we assess usability for specific telehealth functionalities, such as synchronous and asynchronous use (eg, videoconferencing, emailing, and remote monitoring). Assessing these specific tools is crucial in determining which telehealth functionalities work best across different economic settings as requirements likely differ among HICs, HMICs, and LMICs.

### Conclusions

Since the onset of the COVID-19 pandemic, telehealth has flourished and emerged as an indispensable resource for improving access to care worldwide, particularly among HCPs treating patients with NCDs. This study demonstrates that telehealth usability is driven by technical infrastructure (connection stability and support information), user profiles (profession), and an “experience effect” derived from specific engagement patterns (frequency and duration of use). Furthermore, this study uncovered notable differences in these patterns across the 4 participating countries. These findings have potential implications for the practice and policy of telehealth systems in HIC, HMIC, and LMIC contexts, highlighting key areas that warrant attention to enhance system usability and ensure effective health care delivery for patients with NCDs worldwide.

## Supplementary material

10.2196/64070 Checklist 1 STROBE checklist.

## Abbreviations

HCP: health care professional
HIC: high-income country
HMIC: high- to middle-income country
LMIC: low- to middle-income country
NCD: noncommunicable disease
STROBE: Strengthening the Reporting of Observational Studies in Epidemiology
SUS: System Usability Scale
